# Comparative Glycomics of Immunoglobulin A and G From Saliva and Plasma Reveals Biomarker Potential

**DOI:** 10.3389/fimmu.2018.02436

**Published:** 2018-10-23

**Authors:** Rosina Plomp, Noortje de Haan, Albert Bondt, Jayshri Murli, Viktoria Dotz, Manfred Wuhrer

**Affiliations:** Center for Proteomics and Metabolomics, Leiden University Medical Center, Leiden, Netherlands

**Keywords:** *N*-glycan, *O*-glycan, saliva, immunoglobulin G, immunoglobulin A, joining chain, secretory component

## Abstract

The *N*-glycosylation of immunoglobulin (Ig) G, the major antibody in the circulation of human adults, is well known for its influence on antibody effector functions and its alterations with various diseases. In contrast, knowledge on the role of glycans attached to IgA, which is a key immune defense agent in secretions, is very scarce. In this study we aimed to characterize the glycosylation of salivary (secretory) IgA, including the IgA joining chain (JC), and secretory component (SC) and to compare IgA and IgG glycosylation between human plasma and saliva samples to gain a first insight into oral cavity-specific antibody glycosylation. Plasma and whole saliva were collected from 19 healthy volunteers within a 2-h time window. IgG and IgA were affinity-purified from the two biofluids, followed by tryptic digestion and nanoLC-ESI-QTOF-MS(/MS) analysis. Saliva-derived IgG exhibited a slightly lower galactosylation and sialylation as compared to plasma-derived IgG. Glycosylation of IgA1, IgA2, and the JC showed substantial differences between the biofluids, with salivary proteins exhibiting a higher bisection, and lower galactosylation and sialylation as compared to plasma-derived IgA and JC. Additionally, all seven *N*-glycosylation sites, characterized on the SC of secretory IgA in saliva, carried highly fucosylated and fully galactosylated diantennary *N*-glycans. This study lays the basis for future research into the functional role of salivary Ig glycosylation as well as its biomarker potential.

## Introduction

Using saliva for diagnostic purposes has gained increasing popularity due to its various advantages over plasma or serum. First, saliva can be easily and painlessly collected from donors without the need for specialized equipment or training. Second, next to proteins derived from serum (ca. 27% of total salivary proteins), saliva also contains locally produced proteins which might reflect diseases that affect the oral cavity, such as Sjögren's syndrome, or oral cancer (1–3). Saliva assays are already available on the market, e.g., for the detection of antibodies against HIV (1). Moreover, numerous potential salivary protein-, DNA-, RNA-, and small-molecule-based biomarkers for various diseases have been proposed, as recently reviewed (4). Furthermore, two studies using global lectin-based profiling of glycans in saliva revealed associations with breast cancer and Sjögren's syndrome (5, 6).

Glycosylation is a prevalent posttranslational modification which heavily influences the structure and function of proteins, as is well known for plasma-derived immunoglobulin (Ig) G. The glycosylation of the fragment crystallizable (Fc) portion of IgG has been shown to influence binding to Fcγ receptors (FcγRs) and complement factors (7). Moreover, plasma IgG glycosylation has been associated with various diseases and could, therefore, be exploited for diagnostic and therapeutic approaches in the future (8–10). In contrast, for other Igs, such as IgA, less is known about the relationship between their glycosylation and effector functions. Although IgA *N*-glycosylation was shown to influence the IgA transport from circulation to mucosal tissue (11) and the *O*-glycosylation of IgA1 is considered a major factor in the pathogenesis of IgA nephropathy (12), the absence of the Fc *N*-glycosylation of IgA1 had no effect on FcαR binding (13). Site-specific investigation of antibody glycosylation in human samples other than plasma has been limited to cerebrospinal fluid and synovial fluid for IgG (14, 15) and colostrum for IgA (16, 17). The glycosylation of saliva-derived IgG and IgA has only been crudely examined using lectin binding assays, and without a direct comparison to blood (18, 19).

**Figure 1:**
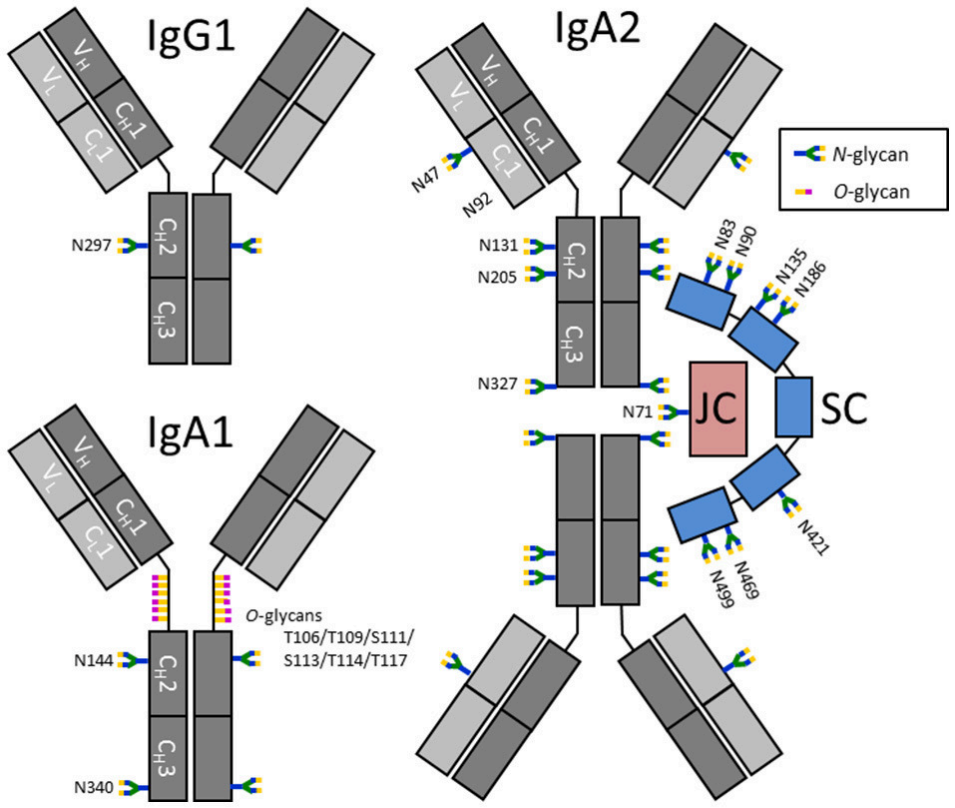
Schematic representation of IgG1, IgA1, and SIgA2 and their glycosylation sites. IgA2 is shown in its dimeric form, in a complex with a joining chain (JC; red) and a secretory component (SC; blue). The JC and SC are covalently linked to IgA by disulfide bonds (not shown). Each Ig monomer is composed of two heavy chains (dark gray) and two light chains (light gray), again connected by disulfide bonds. The chains are further subdivided as constant (C) and variable (V) domains. *N*-glycosylation sites on IgG are indicated by their amino acid number according to the nomenclature used by conventional literature, e.g., (24), while IgA, the JC and SC have UniProt numbering (25). N92 on IgA2 was previously reported to be glycosylated, however, N92 on the IgA2 allotype found in our samples [A2m(1)] was not incorporated in a *N*-glycosylation consensus sequence and was found to be non-glycosylated.

The lack of knowledge about salivary antibody glycosylation is mainly due to the fact that the antibody concentrations are much lower than in plasma, posing a major challenge for their in-depth characterization. Plasma contains approximately 12.5 mg/mL IgG and 2.2 mg/mL IgA, while the concentrations for unstimulated whole saliva are estimated at approximately 0.014 and 0.19 mg/mL for IgG and IgA, respectively (20, 21). Salivary IgG is thought to mainly be derived from circulation, while a minority (<20%) is produced by local plasma cells in gingival lesions or salivary glands (20, 22). In contrast, more than 95% of salivary IgA is produced locally by plasma cells in various glands, where it can form a dimeric complex via the associated joining chain (JC) (23). Secretion of dimeric IgA across the epithelial layer is enabled by the polymeric Ig receptor (pIgR), of which a part remains bound to the IgA and is known as the secretory component (SC). As a consequence, secretory (S)IgA as a complex of dimeric IgA covalently linked to the JC and SC (Figure 1), contributes to more than 80% of the salivary IgA pool, as opposed to plasma IgA which is predominantly (~90%) monomeric (20, 26). In addition, the ratio between IgA1 and IgA2 is dependent on the source of IgA: while saliva contains approximately 35% IgA2, around 20% IgA2 is found in serum (20, 26). In contrast to IgG, which only contains one glycosylation site, or two in case of some IgG3 variants (27), the constant domains of IgA1 and IgA2 contain two up to five potential *N*-glycosylation sites, respectively, and IgA1 carries up to six *O*-glycans in the hinge region (24, 28) (Figure 1). In addition, the JC has one and the SC seven *N*-glycosylation sites (Figure 1). This further complicates detailed (S)IgA glycosylation analysis. However, in recent years great progress has been made with respect to both measurement sensitivity and data analysis tools in the field of glycoproteomics, facilitating site-specific glycosylation analysis of minute amounts of Igs (29–32).

Here, we developed a method for the analysis of IgG and (S)IgA glycosylation in whole saliva using bead-based affinity chromatography purification, followed by tryptic digestion and analysis with nano liquid chromatography (nanoLC)-electrospray ionization (ESI)-quadrupole time-of-flight (QTOF)-mass spectrometry (MS). The method can be used in a 96-well plate format and was applied to characterize and compare the glycosylation of both IgG and (S)IgA in saliva and plasma of 19 healthy individuals. To the best of our knowledge we are the first to report site-specific glycoprofiling of antibodies in saliva.

## Materials and methods

### Collection of biofluids and preprocessing

Nineteen healthy donors (18 Caucasians and 1 of South Asian ancestry) were recruited to donate blood and saliva. The study population included 13 females and 6 males, with an average age of 28.4 years (range: 20–42). All donors gave informed consent, and the study was approved by the Medical Ethics Commission of the Leiden University Medical Center (P16.189). Both biofluids were collected between 10:30 a.m. and 12:30 p.m. on the same day. Donors were instructed to rinse their mouth with water an hour prior to collection of saliva and abstain from food and beverages until sample collection was finished. Saliva was collected by unstimulated drooling into a 5-mL Eppendorf tube for approximately 10 min, and immediately frozen at −20°C. Subsequently, 10 mL of venous blood was collected in an EDTA vial. The blood was centrifuged at 3184 × *g* for 5 min and the clear upper fraction, consisting of plasma, was collected and frozen at −20°C.

Saliva samples underwent a preprocessing step to reduce viscosity: After thawing, the samples were centrifuged at 3184 × *g* at 4°C for 30 min. Four hundred μL of supernatant was collected from each sample and dispersed over four aliquots of 100 μL (for IgG and IgA purification each in duplicate), which were added to a 96-well filter plate with a 10 μm pore frit (Orochem, Naperville, IL). The samples were centrifuged at 200 × *g* for 1 min, and flow-throughs were used for IgG and IgA affinity purification immediately after.

### Purification and digestion of immunoglobulins from donor plasma and saliva

IgG and IgA were each purified in duplicate on separate plates using affinity bead chromatography, based on a protocol described previously (33). For IgG purification, 15 μL of Protein G Sepharose 4 Fast Flow beads (GE Healthcare, Uppsala, Sweden) were added per well on an Orochem filter plate and washed three times with phosphate-buffered saline (PBS). For IgA purification, the same procedure was followed using 2 μL CaptureSelect IgA Affinity Matrix beads (Thermo Fisher Scientific, Breda, The Netherlands). For IgG purification, either 100 μL of saliva or 2 μL of plasma was added to each well, while 100 μL of saliva or 5 μL of plasma was used for IgA purification. Sample volumes were brought to a total of 200 μL by the addition of PBS. The plates were incubated for 1 h while shaking at 750 rpm with 1.5 mm orbit (Heidolph Titramax 100; Heidolph, Kelheim, Germany) to accommodate binding.

Using a vacuum manifold, the samples were washed three times by adding 400 μL PBS, followed by three times 400 μL of purified water (Purelab Ultra, maintained at 18.2 MΩ; Veolia Water Technologies Netherlands B.V., Ede, The Netherlands). The antibodies were eluted from the beads by adding 100 μL of 100 mM formic acid (Sigma-Aldrich, Zwijndrecht, The Netherlands), incubating for 5 min at 750 rpm, and centrifuging at 100 × *g* for 2 min. Samples were dried for 2 h at 60°C in a vacuum centrifuge.

The IgG samples were resolubilized in 20 μL 50 mM ammonium bicarbonate (Sigma-Aldrich) while shaking for 5 min at 750 rpm. Twenty μL of 0.05 μg/μL tosyl phenylalanyl chloromethyl ketone (TPCK)-treated trypsin (Sigma-Aldrich) in ice-cold purified water was added per well, and the samples were incubated at 37°C overnight.

In contrast to IgG, IgA molecules need to be reduced and alkylated prior to digestion to obtain peptides covering all glycosylation sites. IgA samples were resolubilized in 30 μL 30 mM ammonium bicarbonate, 12.5% acetonitrile (Biosolve, Valkenswaard, the Netherlands) while shaking for 5 min at 750 rpm. Subsequently, 5 μL of 35 mM dithiothreitol (Sigma-Aldrich) was added, followed by 5 min incubation at room temperature on a shaker (750 rpm) and 30 min incubation at 60°C in an oven. After cooling to room temperature, 5 μL of 125 mM iodoacetamide (Sigma-Aldrich) was added and the samples were incubated in the dark while shaking for 30 min. Two μL of 100 mM dithiothreitol was added to quench the iodoacetamide. Finally, 8 μL of 0.08 μg/μL TPCK-treated trypsin in ice-cold purified water was added per well, and the samples were incubated at 37°C overnight. Before LC-MS analysis, IgA and IgG samples derived from plasma were diluted twice and 20-times, respectively, with purified water, whereas saliva-derived samples were not diluted. Assuming no losses and prior concentrations of 12.5 mg/mL plasma IgG, 0.014 mg/mL saliva IgG, 2.2 mg/mL plasma IgA and 0.19 mg/mL saliva IgG (20, 21), the concentrations of the final digests were 0.031, 0.035, 0.110, and 0.380 mg/mL for plasma IgG, saliva IgG, plasma IgA, and saliva IgA, respectively.

### Digestion and *N*-glycan release of IgA samples for MS/MS analysis

For the fragmentation analysis by MS/MS of the IgA glycopeptides, a separate purification and tryptic digestion of IgA was done in triplicate on two saliva samples from two donors from the study described above, a pooled-plasma standard from a minimum of 20 human donors (VisuCon-F Frozen Normal Control Plasma; Affinity Biologicals, Ancaster, Canada), 10 μg of a human plasma-derived IgA standard (Lee Biosolutions, Maryland Heights, MO), and a human colostrum-derived SIgA standard (Athens Research and Technology, Athens, GA). The procedure was similar to what is described above, except that 1 μg trypsin per sample was used instead of 0.64 μg. Twenty μL of trypsin-digested sample was collected and heated to 95°C to inactivate the trypsin. After cooling to room temperature, 1 μL of *N*-glycosidase F (Roche Diagnostics, Mannheim, Germany) was added and the samples were incubated at 37°C overnight. The samples obtained from the pooled-plasma standard were diluted twice and the (S)IgA standard samples thrice with purified water, whereas the saliva-derived samples were not diluted.

### NanoLC-ESI-QTOF-MS(/MS) analysis

IgG and IgA samples were present on separate plates and measured on different days. Each pair of plasma and saliva sample of the same donor was analyzed successively, but the duplicate pair of the same donor was measured at a later time point. Blanks consisting of purified water were run after every 20 samples. Two hundred nL of each sample was injected into an Ultimate 3000 RSLCnano system (Dionex/Thermo Scientific, Breda, the Netherlands) coupled to a quadrupole-TOF-MS (MaXis HD; Bruker Daltonics, Bremen, Germany). The LC system was equipped with an Acclaim PepMap 100 trap column (particle size 5 μm, pore size 100 Å, 100 μm × 20 mm, Dionex/Thermo Scientific) and an Acclaim PepMap C18 nano analytical column (particle size 2 μm, pore size 100 Å, 75 μm × 150 mm, Dionex/Thermo Scientific). A mixture of solvent A (0.1% formic acid in purified water) and solvent B (95% acetonitrile) was applied with a constant flow of 0.7 μL/min using a linear gradient: t(min) = 0, %B = 3; t = 5, %B = 3; t = 35, %B = 53; with washing and equilibration starting at t = 36, %B = 70; t = 38, %B = 70; t = 39, %B = 3; t = 58, %B = 3. The sample was ionized in positive-ion mode using a CaptiveSprayer (Bruker Daltonics) electrospray source at 1300 V. A nanoBooster (Bruker Daltonics) was used to enrich the nitrogen gas with acetonitrile to enhance ionization efficiency. Mass spectra were acquired with a frequency of 1 Hz and the MS ion detection window was set at mass-to-charge ratio (*m/z)* 550–1800. Fragmentation spectra were recorded with a detection window of *m/z* 50–2800.

### Data processing

LC-MS(/MS) data were first examined manually using DataAnalysis (Bruker Daltonics). Glycopeptides were identified based on their *m/z* and literature (Supplemental Table S1) (17, 33–40). In the following, glycosylation site numbering for IgG is based on conventions commonly used in literature, e.g., (24), and for IgA1, IgA2, the JC and the SC, UniProt numbering is used (25) (Figure 1). For each glycopeptide cluster, here defined as a group of glycopeptides sharing the same peptide portion, e.g., IgA2 N205, at least one glycopeptide was characterized by MS/MS fragmentation, elucidating the glycan composition and the identity of the peptide (Supplemental Figure S1). In order to provide information on the peptide sequence of the IgA glycopeptides, a proteomics analysis (MASCOT Deamon version 2.2.2; Matrix Science, London, UK) was run on the LC-MS/MS data of the *N*-glycosidase F-digested IgA samples, in which the *N*-glycans had been released. The following settings were used: database: SwissProt (2017_09); taxonomy: *Homo sapiens*; enzyme: trypsin; fixed modifications: carbamidomethyl (C); variable modifications: oxidation (M), deamidated (NQ) and Gln
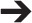
pyro-Glu (N-term Q); maximal number of missed cleavages: 2; peptide tolerance MS: 0.05 Da; peptide tolerance MS/MS: 0.07 Da (Supplemental Table S2). The peptides covering IgA2 N47 and N92 were not recognized by the software, but could still be manually identified (Supplemental Figure S2). The proteomics data are available via ProteomeXchange with identifier PXD011228.

For IgA1/2 N340/N327, two different peptide sequences were identified in *N*-glycosidase F-treated samples: the expected LAGKPTHVNVSVVMAEVDGTCY and the truncated LAGKPTHVNVSVVMAEVDGTC, lacking the C-terminal tyrosine, as described before by Bondt et al. (35). Both of these peptides eluted at two different retention times: a minor peak (<20%) first, and a higher peak eluting 1 min later. The same pattern could be seen in samples that were not treated with *N*-glycosidase F. For data analysis, only the higher, later-eluting peak was included, since the lower peak was often of too low signal quality.

LC-MS data were calibrated in DataAnalysis based on a list of the expected *m/z* values of either IgG1 N297 or IgA N205 glycopeptides. The data files were then converted to mzXML format using the MSconvert program from the ProteoWizard 3.0 suite. Alignment of the time axis of the data and extraction of glycopeptide signal intensities, based on sum spectra, was done using the in-house developed LacyTools software, with a list of manually compiled glycopeptides and their retention times as input (29). For signal extraction, area integration was performed within an *m/z* window of ±0.05 Th around each isotopic peak (with the minimum theoretical % of signal intensity covered by the sum of the integrated isotopic peaks at 85%) within an individually specified time window surrounding the retention time. This resulted in background-corrected signal areas for each glycopeptide per charge state {[M+2H]^2+^ to [M+7H]^7+^}. Whether an analyte was present in a sample, was determined based on the signal-to-noise ratio (S/N; > 9), the isotopic pattern quality score (IPQ; < 0.2) and the mass accuracy (<10 ppm deviation) of each signal per charge state. Data of specific glycopeptide clusters were excluded for further processing when either the total number of analytes detected was <50% of the maximum number detected for that cluster, or when the summed absolute intensity of the detected analytes was <5% of the maximum sum observed for that cluster. This assessment was performed separately for samples obtained from saliva or plasma. Subsequently, individual analytes were subjected to quality control criteria to determine which were of sufficient quality for relative quantification. Glycopeptides in specific charge states were included for relative quantification if their signal showed a S/N > 9, IPQ < 0.2 and an absolute mass error <10 ppm in at least 25% of either all plasma or all saliva samples. Finally, all included charge state signals for the same glycopeptide were summed and absolute abundances were corrected for the fraction of isotopes integrated. The glycopeptide signals were normalized on the total signal intensity per glycopeptide cluster, resulting in the relative quantification of each glycopeptide.

Based on the *N*-glycan monosaccharide compositions, glycoforms were categorized as bisected, high-mannose or hybrid, and, in addition, the number of fucoses, galactoses, and sialic acids was determined (Supplemental Table S1). This was used for the calculation of several glycosylation features per glycosylation site, based on total-area normalized data. Fucosylation represents the number of fucoses per complex-type *N*-glycan, i.e., sum [(complex-type species with n fucoses)^*^n]/sum(complex-type species). Bisection represents the fraction of bisected complex-type *N*-glycans, i.e., the sum of all structures presumed to carry a β1-4-linked *N*-acetylglucosamine (GlcNAc) on the innermost mannose, divided by the sum of all complex-type glycoforms. Galactosylation was calculated as the number of galactoses per complex-type *N*-glycan, i.e., sum [(complex-type species with n galactoses)^*^n]/sum(complex-type species). Sialylation was calculated as the number of sialic acids per complex-type *N*-glycan, i.e., sum [(complex-type species with n sialic acids)^*^n]/sum(complex-type species). Sialylation/Galactose represents the number of sialic acids per galactose on complex-type species, i.e., sialylation divided by galactosylation. High-mannose represents the fraction of high-mannose type *N*-glycans, i.e., sum of all structures where the core is elongated only by mannoses, while hybrid-type glycans represent the structures that are either hybrid-type or have only one antenna. In addition, for the *O*-glycosylation on IgA1, glycosylation features were defined as follows. #HexNAc represents the number of *N-*acetylhexosamines per *O*-glycopeptide, i.e., sum [(species with n *N-*acetylhexosamines)^*^n]. #Hex represents the number of hexoses per *O*-glycopeptide, i.e., sum [(species with n hexoses)^*^n]. #SA represents the number of sialic acids per *O*-glycopeptide, i.e., sum [(species with *n* sialic acids)^*^n]. Hex/HexNAc represents the number of hexoses per *N-*acetylhexosamine, i.e., #Hex divided by #HexNAc. Finally, SA/Hex represents the number of sialic acids per hexose, i.e., #SA divided by #Hex.

### Statistical analysis

From each duplicate pair of samples, the sample with the higher absolute signal intensity per glycopeptide cluster was used for statistical analyses (Supplemental Table S3). The IgG and IgA glycosylation features in plasma and saliva originating from the same donor were compared using the Wilcoxon signed rank test (wilcox.test function from the MASS package) in R [v3.1.2; R Foundation for Statistical Computing, Vienna, Austria (41)] and RStudio (v0.98.1091; RStudio, Inc.; Supplemental Table S4). Correlation analysis was performed between the saliva and plasma samples using the cor.test function from the R stats package (use = pairwise.complete.obs, method = spearman) to obtain the correlation coefficient (rho) and associated *p*-value (Table 1). The Wilcoxon signed rank test and correlation analysis were only performed when data from at least 11 paired (plasma and saliva from the same donor) samples were available, which was the case for IgG1 N297, IgG2/3 N297, IgG4 N297, IgA2 N205, and IgA2 N47. Bonferroni correction was performed to account for multiple testing, resulting in a *p*-value threshold for significance of 0.05/28 = 0.00179. Graphs were created either in Microsoft Excel 2010 or Graphpad Prism 7.

**Table 1:** Correlation of derived glycan traits between saliva and plasma samples as shown by the Spearman's correlation coefficient (rho) and associated *p*-values.

				**Fucosylation**		**Bisection**		**Galactosylation**		**Sialylation**		**Sialic acid/galactose**	
**Protein**	**Site**	**Peptide**	**N_pair_**	**rho**	***p*-value**	**rho**	***p*-value**	**rho**	***p*-value**	**Rho**	***p*-value**	**rho**	***p*-value**
IgG1	N297	EEQYNSTYR	17	0.973	***4.7E-06***	0.877	*< **1E-07***	0.627	0.0084	0.843	***9.7E-07***	0.961	***4.6E-07***
IgG2/3	N297	EEQFNSTFR	17	0.716	***0.0017***	0.936	*< **1E-07***	0.699	0.0025	0.748	***8.4E-04***	0.885	*< **1E-07***
IgG4	N297	EEQFNSTYR	12	NA	NA	0.965	*< **1E-07***	0.706	0.013	0.762	0.0059	0.434	0.16
IgA2	N205	TPLTANITK	16	0.232	0.39	0.474	0.066	−0.0529	0.85	0.191	0.48	0.232	0.39
IgA2	N47	SESGQNVTAR	13	NA	NA	0.412	0.16	−0.242	0.43	0.181	0.55	0.423	0.15

*The numbers of included pairs are listed under “N_pair_.” p-values significant after Bonferroni correction are shown in bold (α = 1.7910^−3^). The correlation for IgA1/2 N144/131 and N340/327, JC N71, the SC N-glycosylation and the IgA1 O-glycosylation was not assessed because this data was present in less than 11 paired samples (plasma and saliva from the same individual)*.

## Results

Paired plasma and saliva samples were collected from 19 healthy donors. From these samples, IgG and IgA were separately purified in duplicate using bead-based affinity chromatography. Samples were trypsin-digested and analyzed by nanoLC-ESI-QTOF-MS. Glycopeptides were identified on the basis of the registered *m/z*, tandem mass spectrometric analyses as well as literature information on immunoglobulin glycosylation (Supplemental Table S1, Supplemental Figure S1, S3). Glycosylation features were calculated for each glycosylation site of IgG, IgA, JC, and SC (Supplemental Table S3).

### IgG *N*-glycosylation

**Figure 2:**
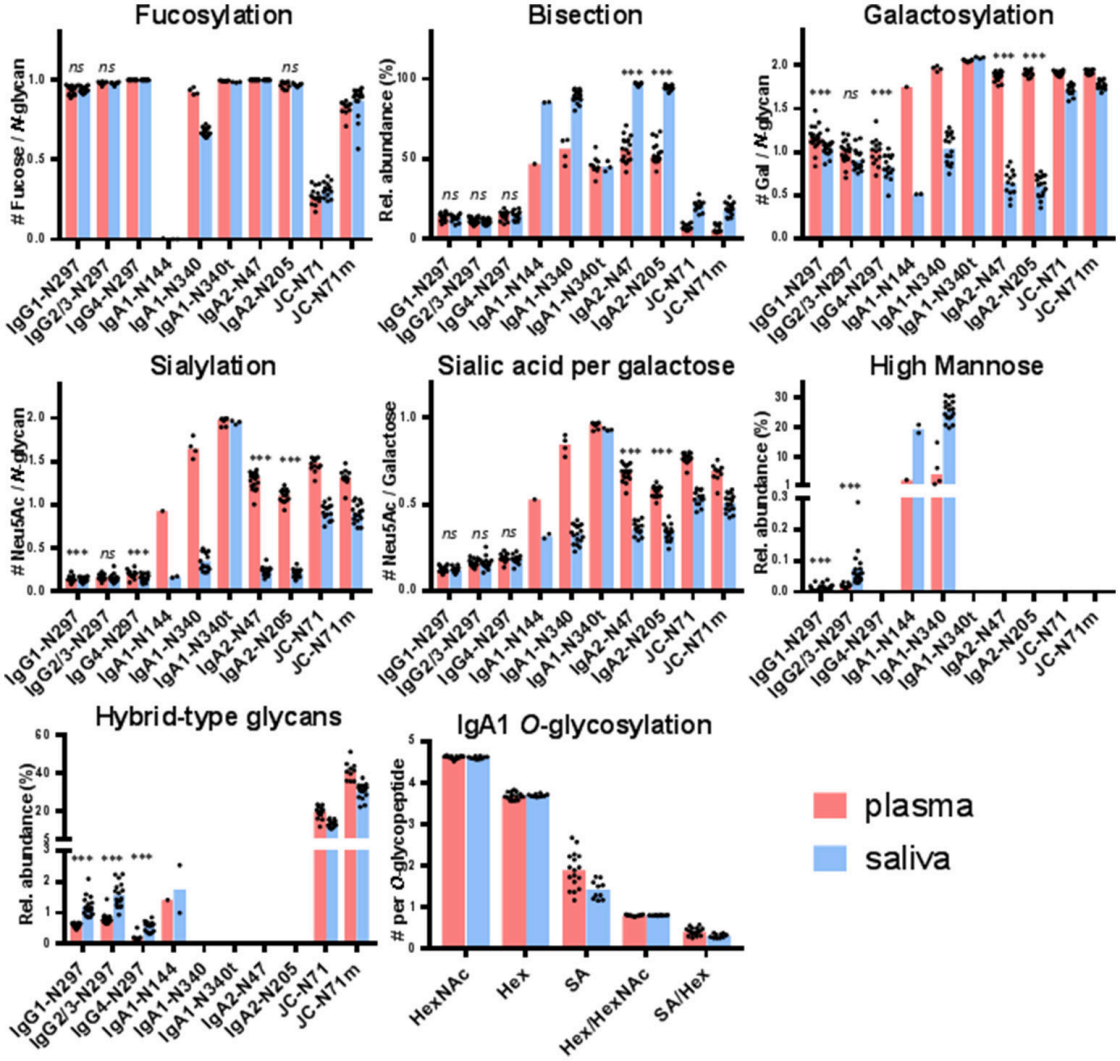
Glycosylation features for each of the glycosylation sites found in IgG, IgA, and the JC. The bar graphs represent the medians per glycosylation site (red for plasma and blue for saliva) and the black dots represent the individual data points for each donor. A Wilcoxon signed rank test was performed between paired samples from plasma and saliva when the total number of pairs was > 10 (Supplemental Table S4). Significant differences are denoted by ^***^, non-significant differences are denoted by *ns*. IgA1-N340t is the *C*-terminally-truncated version of IgA1-N340 and JC-N71m is the miscleavage variant of JC-N71. IgA1-N144 also represents IgA2-N131, and IgA1-N340 and -N340t also represent IgA2-N327 and -N327t.

For IgG1 and IgG2/3 N297, 27 different glycan compositions each were quantified in both the plasma and saliva samples, and 13 for IgG4 N297 (Supplemental Figure S3A, Supplemental Table S1A). IgG1 and IgG4 derived from saliva showed a slightly lower degree of galactosylation and sialylation as compared to plasma (for example, 1.1 and 1.3 times lower medians for IgG1 and IgG4 galactosylation, respectively). In contrast, high-mannose and hybrid-type glycans were more abundant in salivary IgG1 and IgG2/3, as compared to plasma (for example, 2.7 and 3.4 times higher medians for IgG1 and IgG2/3 high-mannose type glycans, respectively; Figure 2, Supplemental Table S4). For IgG1 and IgG2/3, a significant correlation was observed for the fucosylation, bisection, sialylation and sialylation/galactose between plasma and saliva (Table 1), showing the similarities in glycosylation features between the two biofluids to be conserved across the different donors. IgG4 showed similar behavior (Table 1).

### IgA *N*-glycosylation

For IgA2 N205 and N47, 15 and 10 different glycoforms were quantified, respectively, in both the saliva and the plasma samples (Supplemental Figure S3B, Supplemental Table S1B). The *N*-glycans were of the complex-type and mainly diantennary (>98% of total abundance). Two to five-fold differences were observed in the relative abundances of the different glycan types present on plasma- vs. saliva-derived IgA2: salivary *N*-glycans at both N205 and N47 showed a higher degree of bisection and a lower degree of galactosylation, sialylation, and sialylation/galactose (Figure 2, Supplemental Table S4). None of the tested glycosylation traits on IgA2 showed a significant correlation between plasma and saliva samples (Table 1). Of note, for IgA2 *N*-glycosylation site N47 a truncated glycopeptide, i.e., (W)SESGQN_47_VTAR(N), was registered. This peptide features a tryptic *C*-terminal cleavage while the *N*-terminus stems from a chymotryptic cleavage site. The predicted tryptic peptide (K)VFPLSLDSTPQDNVVVACLVQGFFPEPLSVTWSESGQN_47_VTAR(N) was not observed.

The glycopeptides with the peptide moieties LAGKPTHVNVSVVMAEVDGTCY and LAGKPTHVNVSVVMAEVDGTC (truncated; t) were assigned to both IgA1 and IgA2 covering the glycosylation sites N340 and N327, respectively, as these peptide portions are common to both IgA subclasses (Supplemental Table S2). The previously reported sequence variant of IgA2 N327, MAGKPTHIN_327_VSVVMAEADGTC(Y), was not observed in our samples, also not with the naturally occurring polymorphisms I326V and A335V (17, 42, 43). The absolute MS signals of the truncated variant were higher in plasma samples, while those relating to the sequence including the *C*-terminal tyrosine were higher in saliva (Supplemental Table S3). Twenty-one and eight different glycoforms could be quantified on the full-length and the truncated sequence, respectively (Supplemental Table S1B). Furthermore, trends of lower fucosylation (1.4 times lower), galactosylation (2 times lower) and sialylation (5 times lower) and higher bisection (1.7 times higher) in saliva as compared to plasma were observed for IgA1/2 N340/327, but not for IgA1/2 N340/327t (Figure 2, Supplemental Table S4).

IgA1 N144 and IgA2 N131 share the same tryptic glycopeptide sequence LSLHRPALEDLLLGSEAN_144/131_LTCTLTGLR. While nearly all samples failed to provide data of sufficient quality to derive a glycosylation profile, the three samples which did pass data curation (one from plasma and two from saliva, not paired) showed up to 20% high-mannose type glycans and up to 3% hybrid-type/mono-antennary glycans, in addition to the complex-type *N*-glycans (Figure 2). In contrast to other IgA *N*-glycosylation sites, glycans at N144/131 were almost entirely afucosylated (<1% fucosylation; Supplemental Tables S3, S4).

For IgA2 N92, in all samples only non-glycosylated peptides with the sequence HYTN_92_PSQDVTVPCPVPPPPPCCHPR [allotype A2m(1)] were observed. Moreover, samples treated with *N*-glycosidase F did not show deamidated forms of this peptide, indicating that no glycosylated variants of N92 were present prior to *N*-glycosidase F digestion (Supplemental Table S2).

### IgA1 *O*-glycosylation

The potential *O*-glycosylation sites located within the IgA1 hinge region were part of one large tryptic peptide: HYTNPSQDVTVPCPVPSTPPTPSPSTPPTPSPSCCHPR. Forty-two *O*-glycopeptides were quantified. The collective glycan composition consisted of two to five hexoses, three to six HexNAcs and zero to five *N*-acetylneuraminic acids, likely distributed over three to six *O*-glycans (Supplemental Table S1B). Only a minor trend of lower (1.4 times lower) salivary *O*-glycan sialylation was observed as compared to plasma (Figure 2).

### Joining chain (JC) *N*-glycosylation

The single *N*-glycosylation site N71 at the JC was observed on two tryptic peptides: EN_71_ISDPTSPLR (JC N71) and the miscleaved IIVPLNNREN_71_ISDPTSPLR (JC N71m). In general, this glycosylation site contained a higher fraction of monoantennary and hybrid-type glycans than the IgA constant domain *N*-glycosylation sites (between 20 and 50%; Supplemental Table S1C). Furthermore, for this site similar differences between saliva and plasma were observed as for the IgA1 and IgA2 heavy chain *N*-glycosylation sites, namely a higher bisection (3.2 times higher) and lower galactosylation (1.1 times lower) and sialylation (1.7 times lower) in the saliva-derived samples (Figure 2, Supplemental Table S4). The miscleaved glycopeptides showed a higher abundance of fucosylation as compared to the expected tryptic glycopeptides (3.2 times higher in plasma and 2.9 times higher in saliva; Figure 2).

### Secretory component (SC) *N*-glycosylation

**Figure 3:**
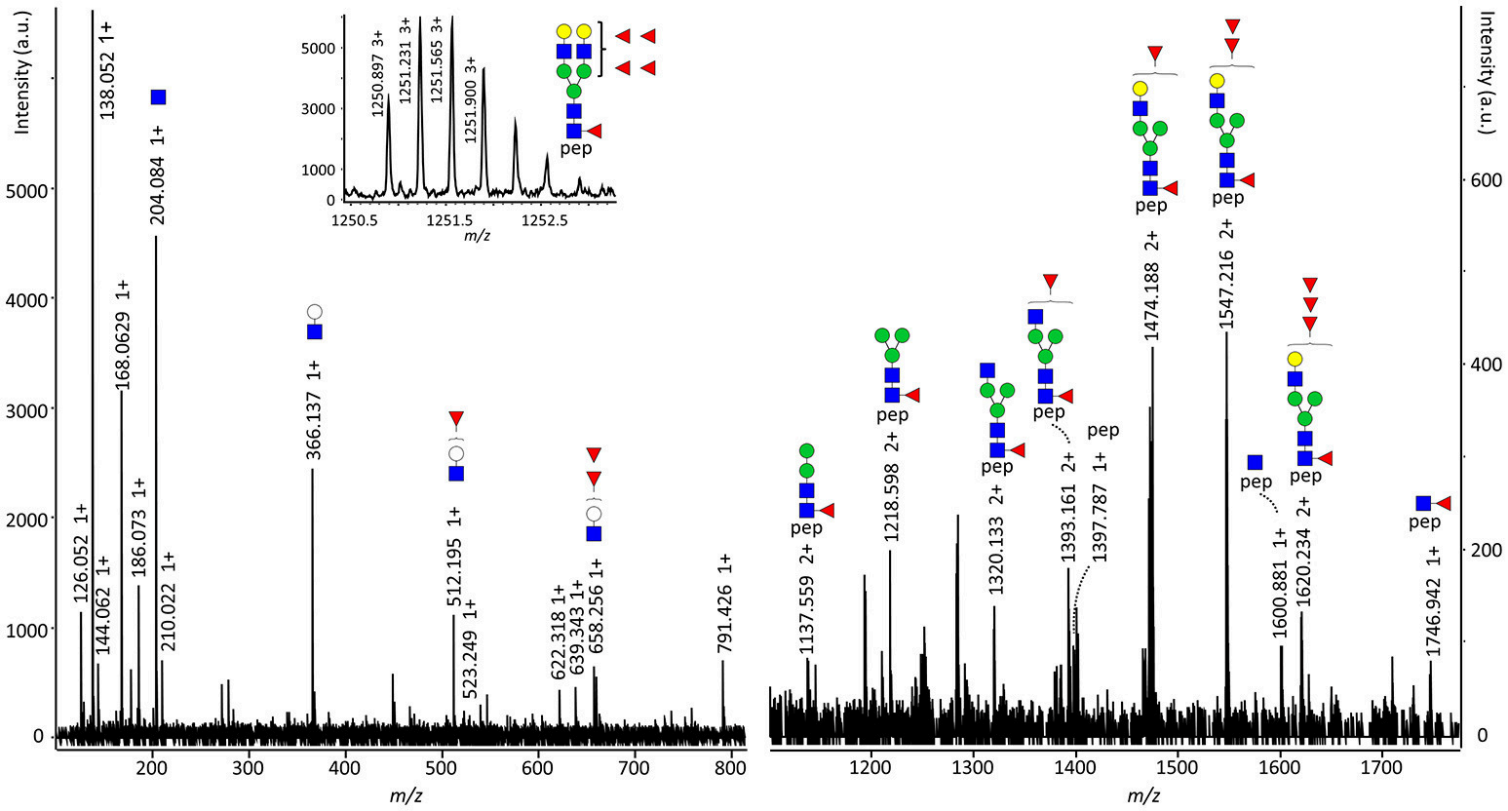
Fragmentation spectrum of SC N469 H5N4F5S0. The parent ions were detected at *m/z* 1250.895 at a retention time of 13.1 min and were assigned as the triply charged variant of [M] = 3749.664 Da, representing SC N469 H5N4F5S0. The structure carries both core and antenna fucosylation, as the former proven by [M+H]^+^ = 1746.942, representing the peptide sequence VPGN_469_VTAVLGETLK plus a GlcNAc and a fucose, and the latter by [M+H]^+^ = 512.195 and 658.256, representing the antenna oxonium ions with one or two fucoses, respectively. pep: VPGN_469_VTAVLGETLK, white circle: hexose, green circle: mannose, yellow circle: galactose, blue square: *N*-acetylglucosamine, red triangle: fucose.

All seven *N*-glycosylation sites of the SC were detected after tryptic digestion of salivary SIgA. Low-intensity signals of SC glycopeptides were also seen in a few plasma samples (Supplemental Table S3). *N*-glycans at N135, N186, N421, and N469 were determined to be complex-type and diantennary, furthermore the antennae were fully galactosylated and partially sialylated (Supplemental Table S1D, Supplemental Figure S4). In addition, on N499, mono-antennary species were identified. The observed glycoforms carried zero to five fucoses, and tandem mass spectrometry indicated the presence of both core and antennary fucosylation (Figure 3, Supplemental Figure S1). On N135, N469, and N499 between 1 and 4% bisection was observed (Supplemental Figure S4). The glycosylation sites N83 and N90 were present on the same tryptic peptide, and the joint glycan composition H_10_N_8_F_2−8_S_0−3_ indicated that the glycans at these two sites were similar to those on other SC sites.

## Discussion

Here we present the first site-specific glycosylation analysis of antibodies in human saliva. IgG and IgA were purified from the plasma and saliva of 19 healthy individuals, and analyzed by LC-MS(/MS). Similar to previous studies on human serum and colostrum IgG or IgA glycosylation which used a comparable analytical methodology (17, 34, 35, 39), we observed glycopeptides covering the *N*-glycosylation sites of IgG1, 2/3 and 4 at N297, IgA1 at N340 and N144, IgA2 at N327, N205 and N131, and of the IgA-associated JC at N71, and the *O*-glycosylation sites in the IgA1 hinge region. We report several non-sialylated glycoforms on the JC that have not been described previously. Moreover, in both plasma and saliva samples we characterized the glycosylation at IgA2 *N*-glycosylation site N47, which had not extensively been described in literature (17, 39). Finally, we provide an overview of the *N*-glycans at all 7 glycosylation sites of the SC in saliva including hitherto unreported molecular species, such as glycoforms with four or five fucoses.

The region surrounding IgA2 N92 is polymorphic and can produce two types of tryptic peptides, HYTN_92_PSQDVTVPCPVPPPPPCCHPR [allotype A2m(1)] and HYTN_92_SSQDVTVPCR [allotype A2m(2)] (44). Since we detected the tryptic IgA2 peptide HYTN_92_PSQDVTVPCPVPPPPPCCHPR in saliva and plasma samples from all individuals included in our study, we conclude that all carry at least one allele of IgA2 allotype A2m(1). This was expected, given that this allele has a frequency of 0.985 in the Caucasian population (44, 45). Allotype A2m(2) peptides were not observed in our samples. Two previous studies reported the NPS sequence on the A2m(1) allotype to be *N*-glycosylated (16, 46), even though the proline following the N92 in A2m(1) does not match the consensus sequence for *N*-glycosylation. Here, however, we found that IgA2 N92 was not glycosylated in either plasma or saliva, in accordance with a previous report on colostrum SIgA (17). In future research also donors expressing the A2m(2) allotype should be included to obtain a complete characterization of IgA glycosylation.

In the current study, we did not detect the (glyco)peptides covering IgA2 N327 when searching for the expected tryptic peptide sequence obtained from UniProt entry IGHA2_HUMAN (P01877) or its naturally occurring variants. However, instead of the methionine in position 319 on IgA2 (25), a leucine was previously reported in allotype A2m(1) (42, 43), resulting in identical peptide sequences for both IgA2 N327 and IgA1 N340. Our observation that all individuals studied expressed IgA2 allotype A2m(1), made us conclude that the glycopeptides that we detected with peptide sequences LAGKPTHVNVSVVMAEVDGTCY and LAGKPTHVNVSVVMAEVDGTC [truncated (35, 47)] represented a mixture of IgA1 N340 and IgA2 N327.

The two differentially cleaved peptide sequences covering N71 of the JC showed a large variation in their glycan fucosylation, with the miscleaved variant having about 3 times higher fucosylation than the expected peptide. This indicates that the core fucose might interfere with tryptic cleavage at R69. Similar differences in fucosylation were found previously (17).

Saliva collection has multiple advantages over the collection of plasma, for example, it is painless and does not require special instrumentation or trained personnel. Specialized devices for saliva collection making use of swabs or chewing on paraffin wax do exist (1, 20). However, we utilized the simplest method, i.e., unassisted, unstimulated drooling, and found that this was sufficient to obtain IgG and IgA for a thorough glycomic analysis. A limitation of the current method might be that only solubilized antibodies were obtained, while the fraction associated to, for example, mucins was precipitated during sample preparation. Furthermore, before saliva can be used as a sample for diagnostics, further research is necessary to evaluate robustness in terms of sample collection mode, time point and sample storage. Finally, although the current method can be performed in a 96-well plate format and has the potential to be automated, for its application in a routine laboratory or diagnostic setting further technological development is required that allows the specific quantification of glycoforms of interest, identified in the discovery phase. Despite the mentioned limitations, using saliva Ig glycosylation might have potential in medical research in two different contexts: (i) as a proxy for plasma Ig glycosylation reflecting systemic conditions, and (ii) as a biomarker (panel) or therapeutic target for local perturbations in the oral cavity.

### Saliva Ig glycosylation as proxy for plasma Ig glycosylation

The glycosylation of, in particular, IgG as the most abundant Ig in plasma has been proposed as a biomarker for various physiological and pathological states in multiple large-scale studies (36, 48, 49). Similarly, IgA1 *O*-glycosylation associates with IgA nephropathy, and IgA1 *N*- and *O*-glycosylation showed to alter with pregnancy and rheumatoid arthritis (12, 35, 50). The vast majority of salivary IgG is thought to originate from plasma, and thus differences in glycosylation between plasma- and saliva-derived IgG likely originate from the minority (<20%) of locally produced IgG (20, 22). Here we found salivary IgG at N297 to exhibit a slightly lower galactosylation and sialylation, and a slightly higher abundance of high-mannose and hybrid-type glycans as compared to plasma. However, most IgG glycosylation features, except galactosylation, correlated well between plasma and saliva. This indicates that these features have potential as plasma IgG proxies and, thus, as biomarkers for the systemic health status. Low galactosylation and sialylation of plasma IgG are associated with inflammation and various autoimmune diseases (51, 52). Additionally, galactosylation and sialylation are known to influence the binding of IgG to FcγRs and the C1q protein (7, 53). This suggests that the slight IgG glycosylation differences we found between the two biofluids from healthy individuals may arise from the locally higher inflammatory state in the oral cavity, since it is constantly exposed to pathogens. This aspect should be taken into account when further exploring the use of salivary IgG glycosylation as biomarker for systemic diseases, with special attention regarding possible co-occurring oral inflammatory conditions, such as periodontitis (gum disease). In contrast to IgG, the *N*-glycosylation of IgA was not correlated between plasma and saliva in our study. This is likely due to either the different origin of plasma cells producing the two pools of IgA proteins or to a difference in processing, such as dimerization and J-chain binding, or their translocation through the epithelial cell layer. Our findings in healthy individuals aged between 20 and 42 years suggest that salivary IgA cannot be used as a proxy for plasma IgA *N*-glycosylation.

### Saliva Ig glycosylation as a biomarker (panel) or therapeutic target for local perturbations in the oral cavity

Differences of Ig glycosylation in locally produced biofluids can be exploited as biomarkers for local pathological processes. Accordingly, IgG glycosylation in cerebrospinal and synovial fluid from multiple sclerosis and rheumatoid arthritis patients, respectively, revealed biofluid-specific associations with disease (14, 15). In addition, patients with advanced periodontitis were reported to exhibit a lower salivary IgG galactosylation than healthy individuals (18). Our data suggest that salivary IgA *N*-glycosylation analysis also provides a promising tool to detect local glycosylation perturbations in the oral cavity which might reflect pathological processes. IgA is the most abundant antibody in human saliva, and SIgA is regarded as an important first line of defense against pathogens on mucosal surfaces (11, 20, 21). Interestingly, the *N*-glycosylation profile reported for colostrum-derived SIgA (17) is similar to our data on salivary SIgA, in terms of a high abundance of glycoforms carrying a bisecting GlcNAc, contrary to plasma-derived IgA. This suggests that in healthy individuals, SIgA shares a common *N*-glycosylation profile, whether it originates from the salivary or mammary glands, but differs from the IgA glycosylation profile in circulation. This might be related to the fact that both salivary and milk SIgA enter the gastrointestinal tract via the same route, and can exert similar functions while passing the oral cavity and the gastrointestinal tract.

Salivary IgA and JC showed a considerably higher bisection and lower galactosylation and sialylation at all observed sites of *N*-glycosylation, except for the truncated variants of IgA1 N340 and IgA2 N327 lacking the *C*-terminal tyrosine, which had a similar glycosylation profile in plasma and saliva. Moreover, the truncated version was low-abundant in saliva and highly abundant in plasma. Therefore we hypothesize that the truncated form originates from circulation and is present in saliva due to leakage or transport from circulation (22), while the full-length variant might mainly be produced and secreted locally in the oral cavity. The majority of salivary IgA and JC is produced locally in plasma B cells of the glandular stroma (20, 26). This suggests that changes in the glycosylation machinery of specifically these cells are the cause for the substantial glycosylation differences between plasma and saliva. For example, an upregulation of beta-1,4-*N*-acetylglucosaminyltransferase III (GnT-III/MGAT3) and a downregulation of beta-1,4-galactosyltransferase (B4GalT) and beta-galactoside-alpha-2,6-sialyltransferase (St6Gal) 1 in the glandular stroma B cells could be responsible for the observed differences in IgA *N*-glycosylation. Alternatively, the pIgR-enabled transport of IgA across the epithelium, the dimerization of IgA, and the binding of the J-chain might be selective for certain glycoforms of IgA. However, for the binding to the pIgR this appears unlikely taking into account a previous study which reported that the removal of either the *N*- or *O*-glycans of polymeric IgA had no influence on pIgR-mediated transcytosis (54). An additional explanation for the differences between plasma and salivary IgA glycosylation might be the presence of glycosidases from oral microbiota in saliva that can alter the glycosylation after SIgA secretion (55). Finally, it could be speculated that asialoglycoprotein receptors, which are located primarily in the liver, could alter the overall glycosylation of plasma IgA by specifically removing non-sialylated proteins from circulation (56), thus leading to a higher level of sialylation compared to saliva. However, this would not explain the differences in the levels of bisection between plasma and saliva. The functional consequences of differences in IgA *N*-glycosylation are at this point not well understood: The *N*-glycans on the constant region of IgA do not seem to be essential for FcR binding, in contrast to the ones on IgG (13). However, IgA sialylation has been shown to be required for dectin-1-mediated transport of SIgA across the epithelium of intestinal cells (11). Furthermore, in addition to the Fab-binding sites, SIgA fucosylation and sialylation enable the binding of the antibody to pathogenic bacteria, acting as a decoy to prevent bacterial adhesion to the epithelium and subsequent infection (57–59). Finally, the *C*-terminal *N*-glycan sialic acids have recently been reported to directly inhibit sialic acid-binding viruses like influenza (60).

The fact that we did not find differences between salivary and plasma IgA1 *O*-glycosylation, except for a slight trend of lower sialylation in saliva, is likely due to their inherently different biosynthetic pathways as compared to *N*-glycans, involving distinct glycosyltransferases (61). Additionally, we here evaluated all *O*-glycosylation sites simultaneously at the same glycopeptide, which provides less resolution than analysis on the single-structure level. Thus, it is difficult to judge on the usefulness of IgA *O*-glycosylation as a potential diagnostic biomarker based on our data. However, future investigations should further assess IgA *O*-glycosylation in a specific disease context, since in plasma it has been associated with pathologies, such as IgA nephropathy and Sjögren's syndrome (12, 62, 63).

A comparison of the salivary IgA SC with plasma was not possible, due to insufficient signal intensities of the plasma SC from most individuals in our study, most likely due to its low abundance in plasma. *N*-glycans on the SC have been shown to be crucial for various functional aspects of SIgA (59). For example, SC glycosylation enables the innate protection against mucosal pathogens (64). Furthermore, SC glycans are essential for a correct localization of IgA in the mucosal lining, resulting in protection from bacterial infection in the respiratory tract of mice (65). Because of the important and diverse role of SC glycosylation, future studies should examine whether salivary SC glycosylation is associated with, for example, periodontitis, to explore its potential as a diagnostic biomarker or therapeutic target.

### Conclusions

Large differences were found between the glycosylation of plasma- and saliva-derived IgA and its JC, while this was not the case for IgG. This suggests that SIgA, locally produced by plasma B cells in the glandular stroma, differs in glycosylation from IgA of the circulation, that presumably is largely produced by circulating plasma cells, pointing toward distinct regulatory mechanisms. An alternative explanation is that SIgA dimerization or transport into the oral cavity might be glycoform-specific, or that certain IgA-producing B cells, expressing a specific glycosylation repertoire, may be relocated to the glandular stroma after activation. The observed differences between plasma and salivary IgA make biofluid-specific analysis of SIgA glycosylation (including the JC and SC) a promising tool for mucosal disease-related biomarker research. IgG glycosylation, on the other hand, showed a good correlation between plasma and saliva in healthy individuals, indicating that salivary IgG might be a proxy to study plasma IgG in situations where a healthy oral environment can be assumed.

## Data availability statement

The raw data supporting the conclusions of this manuscript will be made available by the authors, without undue reservation, to any qualified researcher. The mass spectrometry proteomics data have been deposited to the ProteomeXchange Consortium via the PRIDE (66) partner repository with the dataset identifier PXD011228.

## Author contributions

RP, VD, and MW designed the study. JM and RP performed sample preparation and experimental analysis. RP and NdH processed data, which were further analyzed by RP, NdH, AB, VD, and MW. NdH and RP drafted the manuscript, which was revised by all authors. All authors approved the final manuscript.

### Conflict of interest statement

The authors declare that the research was conducted in the absence of any commercial or financial relationships that could be construed as a potential conflict of interest.
