# Supplementary material for: Comparative Glycomics of Immunoglobulin A and G From Saliva and Plasma Reveals Biomarker Potential
Source: Front Immunol. 2018 Oct 23;9:2436. doi: 10.3389/fimmu.2018.02436 (PMC6206042; doi:10.3389/fimmu.2018.02436)
Supplement: Supplementary file 2 [file Presentation_1.PDF]

Supplementary Material

Comparative Glycomics of Immunoglobulin A and G from Saliva and Plasma Reveals Biomarker Potential

Rosina Plomp[1,#], Noortje de Haan[1,#,*], Albert Bondt[1], Jayshri Murli[1], Viktoria Dotz[1], Manfred Wuhrer[1]

*** Correspondence:** Noortje de Haan, n.de_haan@lumc.nl

[#] Authors contributed equally

Supplementary Figures

Supplemental Figure S1. Annotated LC-ESI-QTOF-MS/MS spectra of a glycopeptide for each glycopeptide cluster of IgA/JC/SC.

Supplemental Figure S2. NanoLC-ESI-IT-MS/MS of two *N*-glycosidase F-treated IgA2 peptides which were not identified by automated proteomics searches.

Supplemental Figure S3. Representative mass spectra for **A**) IgG1 and **B**) IgA2 N205 in plasma and saliva.

Supplemental Figure S4. Derived glycosylation traits for each of the *N*-glycosylation sites found on the secretory component (SC).

Supplementary Tables

Supplemental Table S1. An overview of all glycopeptides which were observed for A) IgG, B) IgA1 and IgA2, C) JC and D) SC.

Supplemental Table S2. An overview of peptides containing potential *N*-glycosylation sites which were identified in tryptic *N*-glycosidase F-treated samples of (S)IgA samples by automated proteomics software MASCOT.

Supplemental Table S3. The glycosylation features and profiles of each donor for plasma and saliva.

Supplemental Table S4. Comparison of derived glycosylation traits of IgG and IgA glycopeptides from paired saliva and plasma samples.

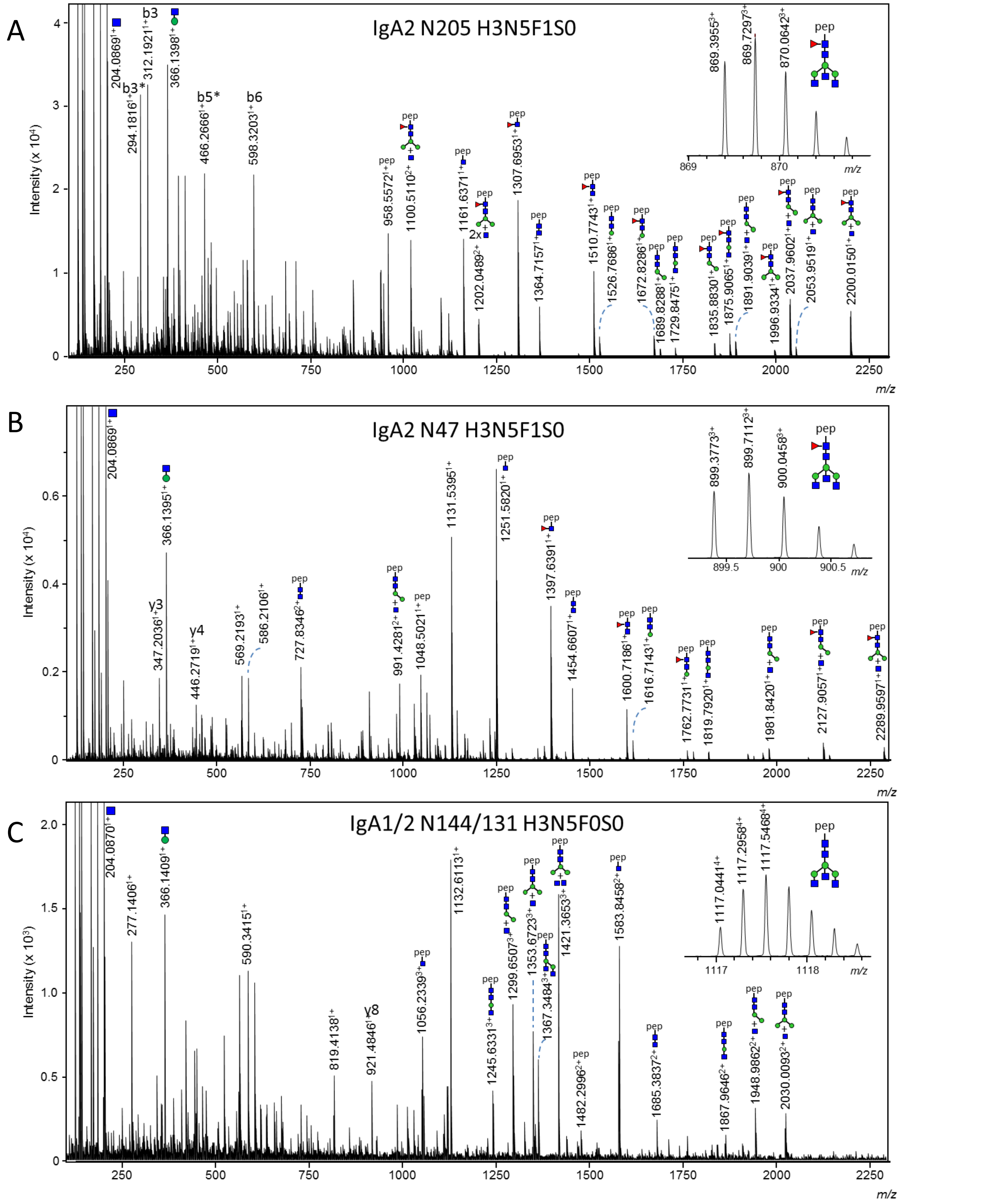

Supplemental Figure S1. Annotated LC-ESI-QTOF-MS/MS spectra of a glycopeptide for each glycopeptide cluster of IgA/JC/SC. The MS1 precursor peak which was fragmented is shown in the upper right corner, together with a schematic depiction of the glycopeptide. Peaks representing glycopeptide or glycan fragments are shown with the relevant glycan structure, and peaks representing peptide fragments are denoted with the y- or b-ion number. Structures showing (partial) loss of one antenna are not meant to show information on which antenna is affected. Water loss is denoted by an asterisk. Blue square: *N*-acetylglucosamine, yellow square: *N*-acetylgalactosamine, green circle: mannose, yellow circle: galactose, white circle: hexose, red triangle: fucose, pink diamond: sialic acid. The mass spectra were derived from the following samples: a saliva sample from one of the donors (**A, B, C, D, F, M, O**), the standard SIgA sample (**G, H, I, J, K, L, N**), and the pooled-plasma sample (**E**).

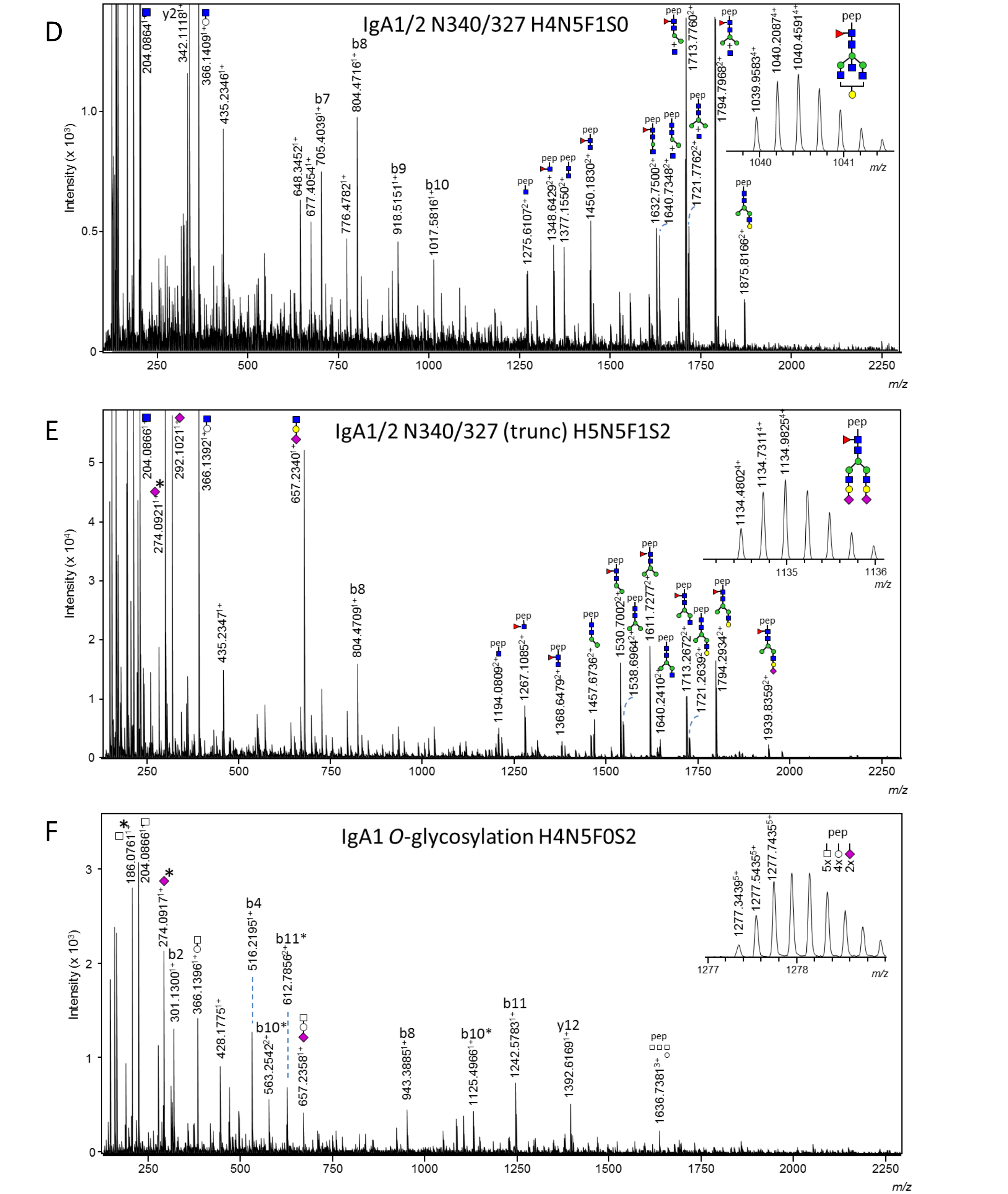

Supplemental Figure S1. Annotated LC-ESI-QTOF-MS/MS spectra of a glycopeptide for each glycopeptide cluster of IgA/JC/SC. The MS1 precursor peak which was fragmented is shown in the upper right corner, together with a schematic depiction of the glycopeptide. Peaks representing glycopeptide or glycan fragments are shown with the relevant glycan structure, and peaks representing peptide fragments are denoted with the y- or b-ion number. Structures showing (partial) loss of one antenna are not meant to show information on which antenna is affected. Water loss is denoted by an asterisk. Blue square: *N*-acetylglucosamine, yellow square: *N*-acetylgalactosamine, green circle: mannose, yellow circle: galactose, white circle: hexose, red triangle: fucose, pink diamond: sialic acid. The mass spectra were derived from the following samples: a saliva sample from one of the donors (**A, B, C, D, F, M, O**), the standard SIgA sample (**G, H, I, J, K, L, N**), and the pooled-plasma sample (**E**).

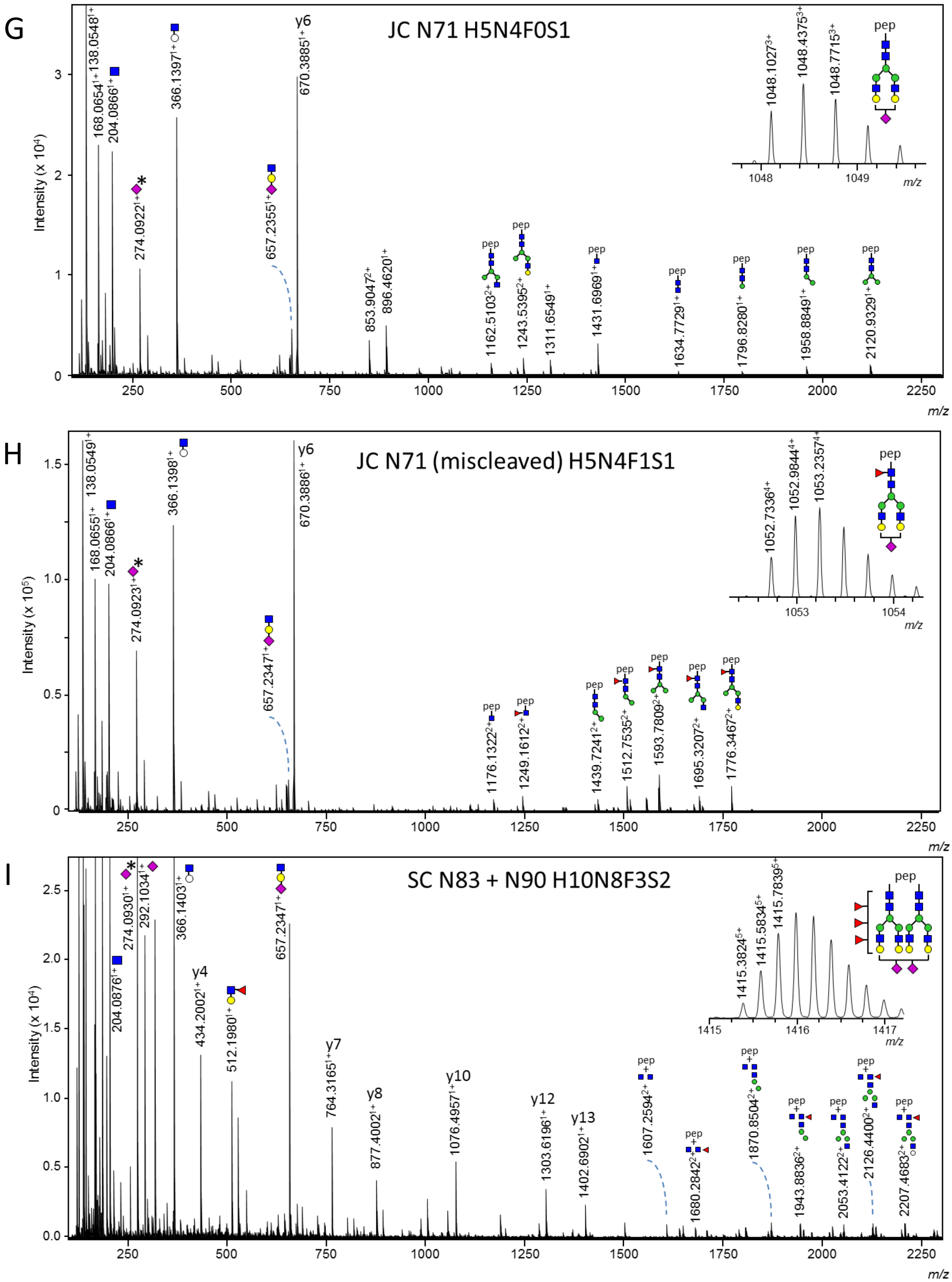

Supplemental Figure S1. Annotated LC-ESI-QTOF-MS/MS spectra of a glycopeptide for each glycopeptide cluster of IgA/JC/SC. The MS1 precursor peak which was fragmented is shown in the upper right corner, together with a schematic depiction of the glycopeptide. Peaks representing glycopeptide or glycan fragments are shown with the relevant glycan structure, and peaks representing peptide fragments are denoted with the y- or b-ion number. Structures showing (partial) loss of one antenna are not meant to show information on which antenna is affected. Water loss is denoted by an asterisk. Blue square: *N*-acetylglucosamine, yellow square: *N*-acetylgalactosamine, green circle: mannose, yellow circle: galactose, white circle: hexose, red triangle: fucose, pink diamond: sialic acid. The mass spectra were derived from the following samples: a saliva sample from one of the donors (**A, B, C, D, F, M, O**), the standard SIgA sample (**G, H, I, J, K, L, N**), and the pooled-plasma sample (**E**).

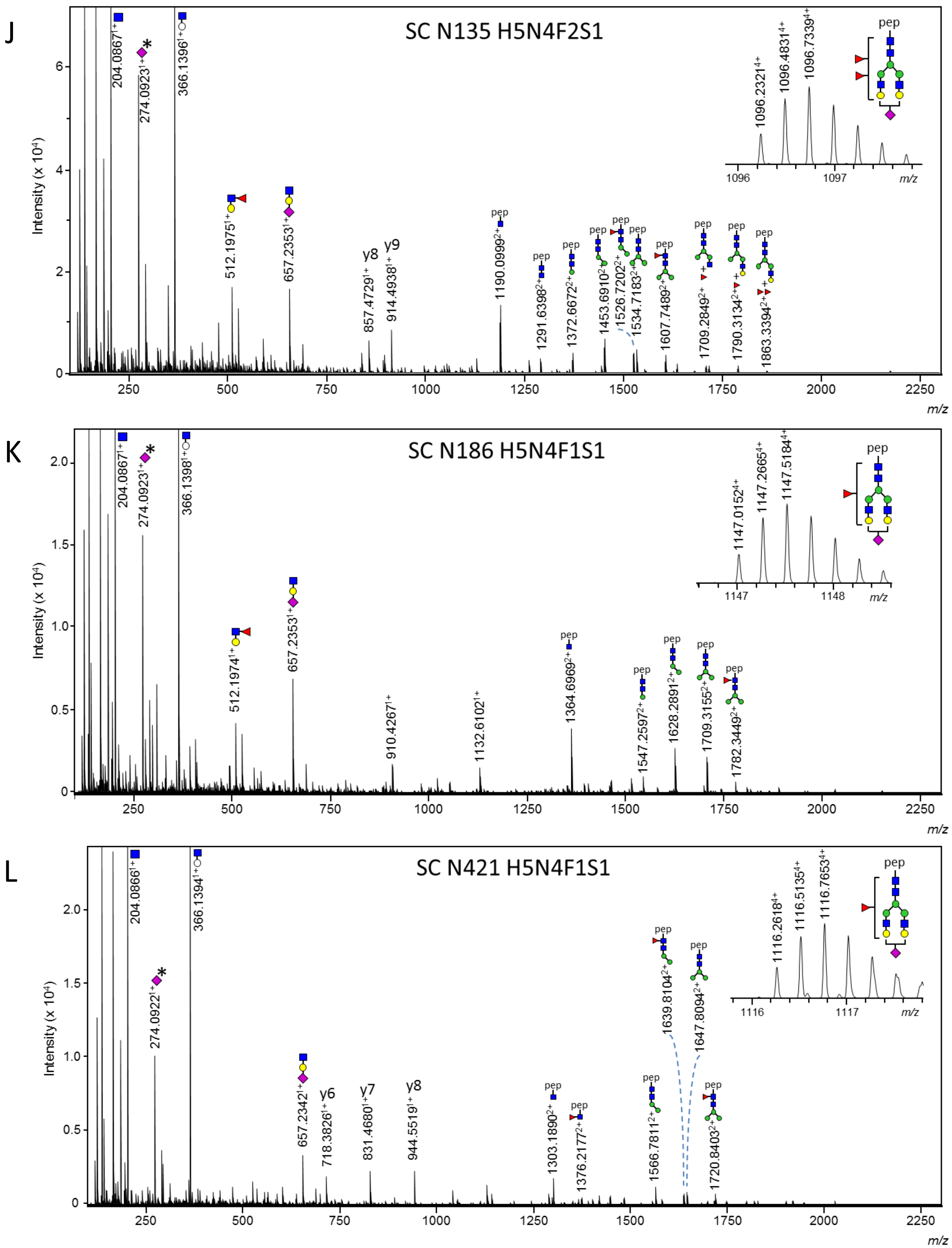

Supplemental Figure S1. Annotated LC-ESI-QTOF-MS/MS spectra of a glycopeptide for each glycopeptide cluster of IgA/JC/SC. The MS1 precursor peak which was fragmented is shown in the upper right corner, together with a schematic depiction of the glycopeptide. Peaks representing glycopeptide or glycan fragments are shown with the relevant glycan structure, and peaks representing peptide fragments are denoted with the y- or b-ion number. Structures showing (partial) loss of one antenna are not meant to show information on which antenna is affected. Water loss is denoted by an asterisk. Blue square: *N*-acetylglucosamine, yellow square: *N*-acetylgalactosamine, green circle: mannose, yellow circle: galactose, white circle: hexose, red triangle: fucose, pink diamond: sialic acid. The mass spectra were derived from the following samples: a saliva sample from one of the donors (**A, B, C, D, F, M, O**), the standard SIgA sample (**G, H, I, J, K, L, N**), and the pooled-plasma sample (**E**).

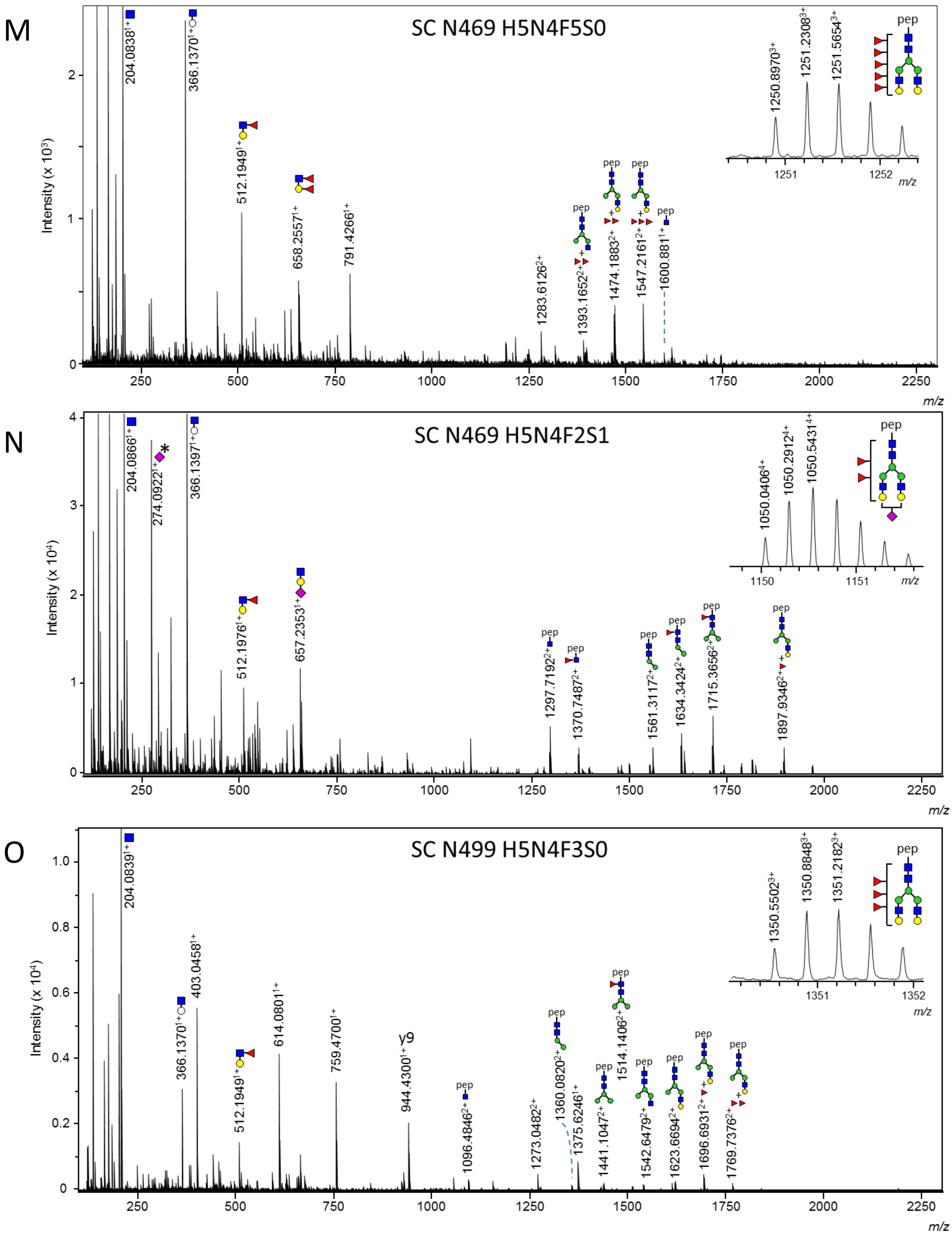

Supplemental Figure S1. Annotated LC-ESI-QTOF-MS/MS spectra of a glycopeptide for each glycopeptide cluster of IgA/JC/SC. The MS1 precursor peak which was fragmented is shown in the upper right corner, together with a schematic depiction of the glycopeptide. Peaks representing glycopeptide or glycan fragments are shown with the relevant glycan structure, and peaks representing peptide fragments are denoted with the y- or b-ion number. Structures showing (partial) loss of one antenna are not meant to show information on which antenna is affected. Water loss is denoted by an asterisk. Blue square: *N*-acetylglucosamine, yellow square: *N*-acetylgalactosamine, green circle: mannose, yellow circle: galactose, white circle: hexose, red triangle: fucose, pink diamond: sialic acid. The mass spectra were derived from the following samples: a saliva sample from one of the donors (**A, B, C, D, F, M, O**), the standard SIgA sample (**G, H, I, J, K, L, N**), and the pooled-plasma sample (**E**).

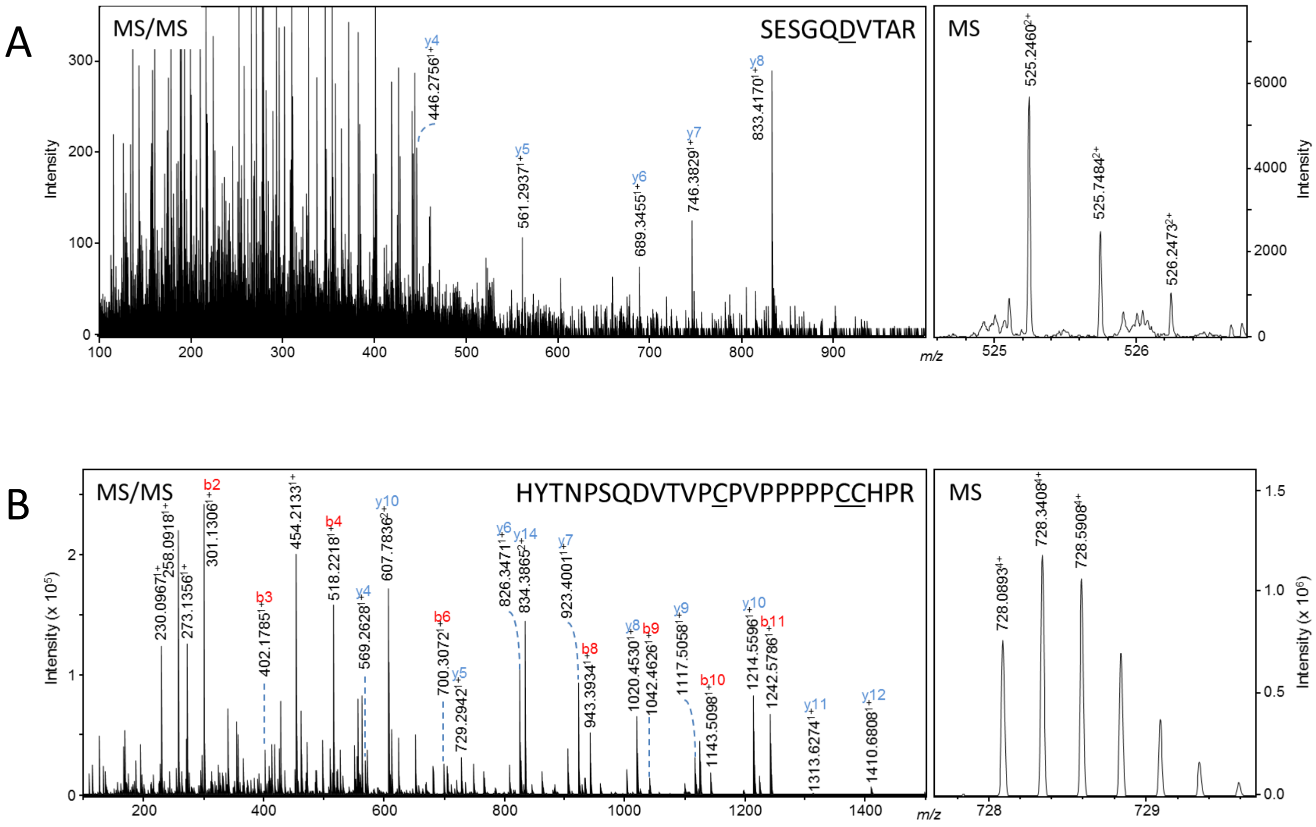

Supplemental Figure S2. NanoLC-ESI-IT-MS/MS of two *N*-glycosidase F-treated IgA2 peptides which were not identified by automated proteomics searches. A) Fragmentation of the deamidated peptide SESGQ(N➔D)VTAR and **B**) the non-deamidated peptide HYTNPSQDVTVPCPVPPPPPCCHPR. The MS1 precursor peak is shown in the panel on the right. Carbamidomethylation is denoted by the underlining of cysteine residues. Spectra shown here were derived from IgA purified from pooled-plasma (**A**) and the SIgA standard (**B**).

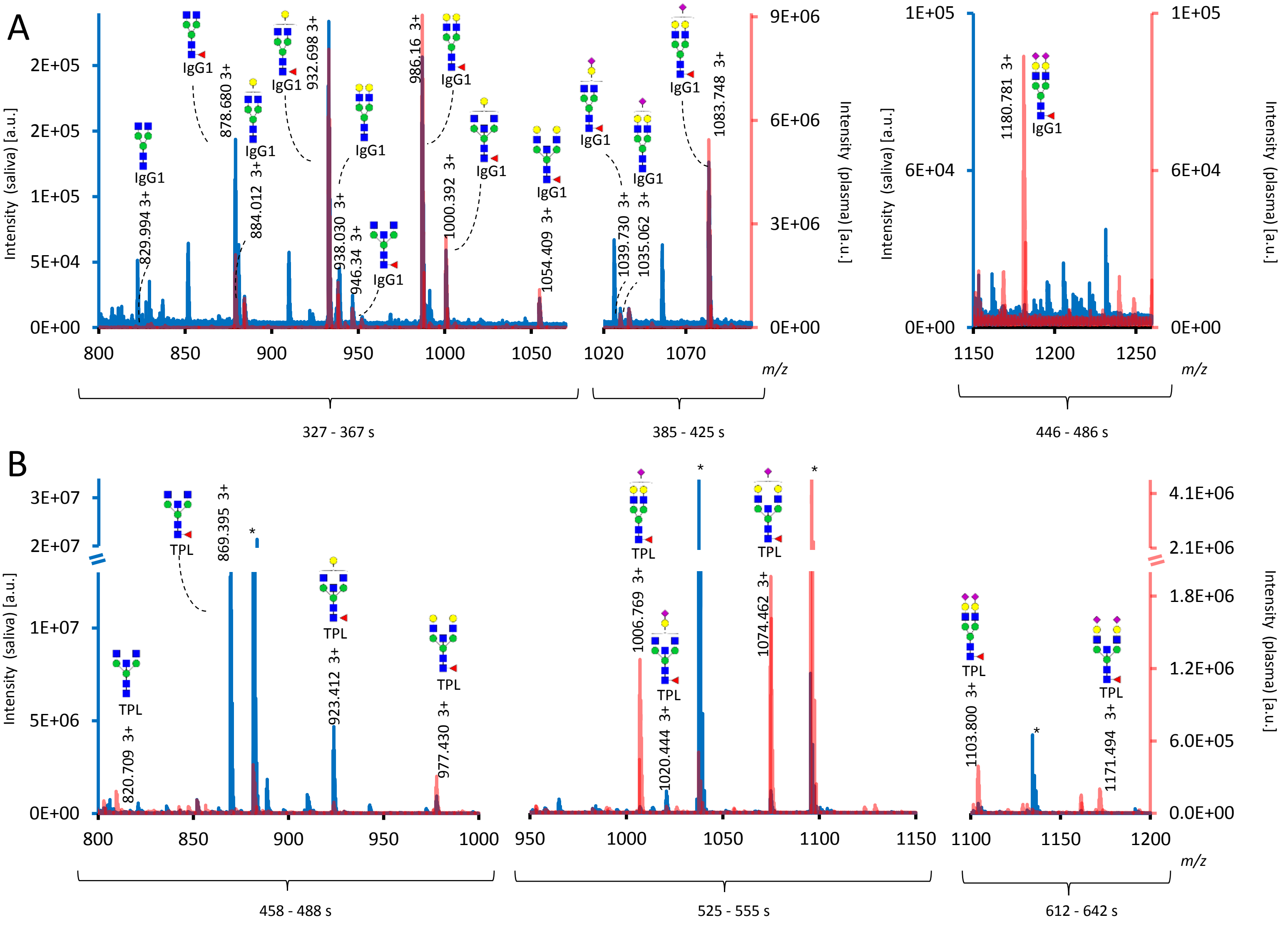

Supplemental Figure S3. Representative mass spectra for A) IgG1 and B) IgA2 N205 in plasma (red) and saliva (blue). IgG1: EEQYN$_{297}$STYR TPL: TPLTAN$_{205}$ITK, green circle: mannose, yellow circle: galactose, blue square: *N*-acetylglucosamine, red triangle: fucoses, pink diamond: *N*-acetylneuraminic acid. Unidentified, non-glycopeptide signals are annotated with an asterisk (*). The retention time ranges under the spectra indicate the ranges over which the mass spectra were summed to create the sum spectra presented.

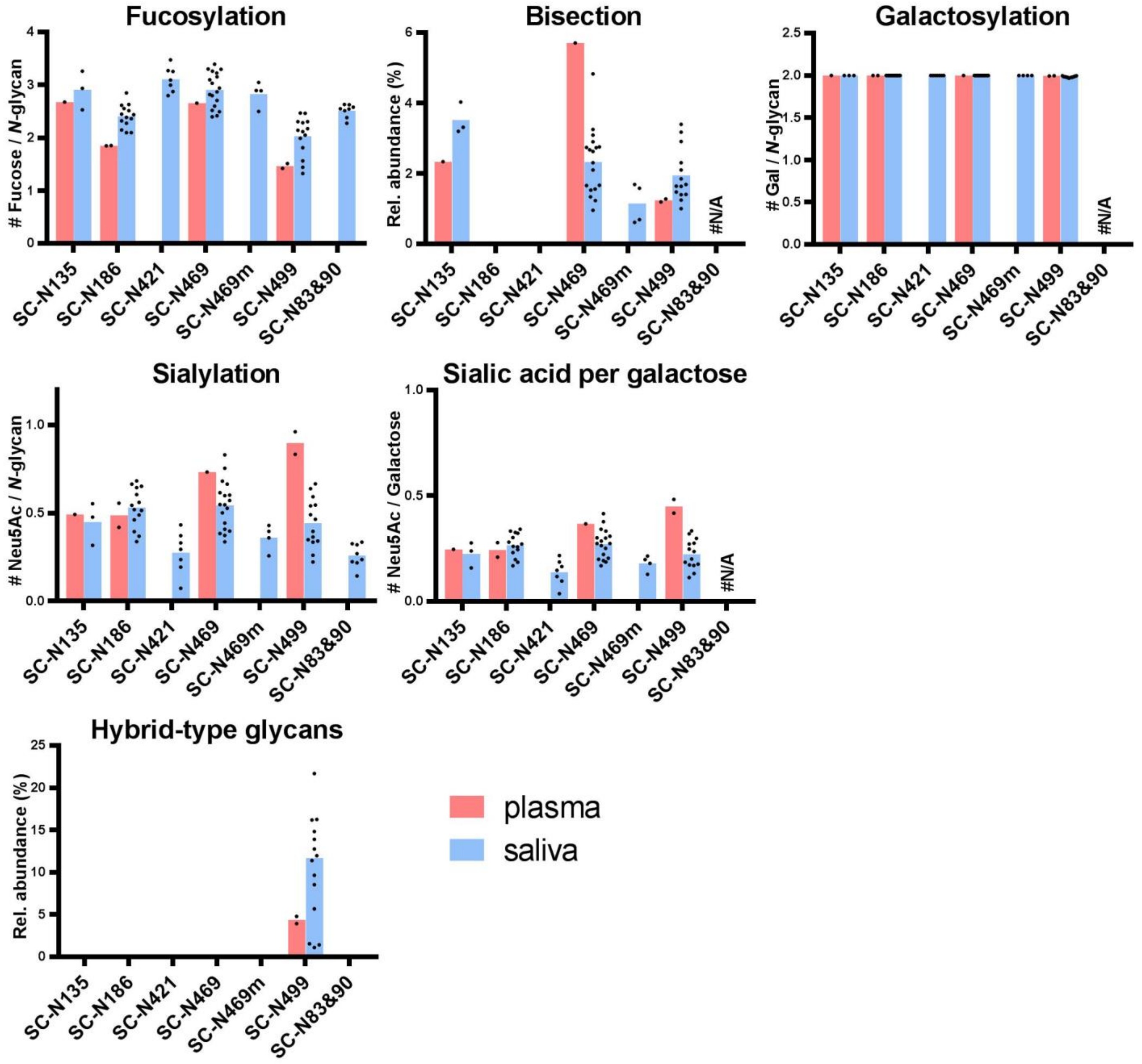

Supplemental Figure S4. Derived glycosylation traits for each of the *N*-glycosylation sites found on the secretory component (SC). The bar graphs represent the medians per glycosylation site (red for plasma and blue for saliva) and the black dots represent the individual data points for each donor. SC-N469m is a miscleavage variant of SC-N469.
